# Lhx2 Determines Odorant Receptor Expression Frequency in Mature Olfactory Sensory Neurons

**DOI:** 10.1523/ENEURO.0230-16.2016

**Published:** 2016-10-31

**Authors:** Guangfan Zhang, William B. Titlow, Stephanie M. Biecker, Arnold J. Stromberg, Timothy S. McClintock

**Affiliations:** 1 Department of Physiology, University of Kentucky, Lexington, Kentucky 40536-0298; 2 Department of Statistics, University of Kentucky, Lexington, Kentucky 40536-0082

**Keywords:** enhancer, gene expression, olfaction, promoter, smell, transcription

## Abstract

A developmental program of epigenetic repression prepares each mammalian olfactory sensory neuron (OSN) to strongly express one allele from just one of hundreds of odorant receptor (OR) genes, but what completes this process of OR gene choice by driving the expression of this allele is incompletely understood. Conditional deletion experiments in mice demonstrate that Lhx2 is necessary for normal expression frequencies of nearly all ORs and all trace amine-associated receptors, irrespective of whether the deletion of *Lhx2* is initiated in immature or mature OSNs. Given previous evidence that Lhx2 binds OR gene control elements, these findings indicate that Lhx2 is directly involved in driving OR expression. The data also support the conclusion that OR expression is necessary to allow immature OSNs to complete differentiation and become mature. In contrast to the robust effects of conditional deletion of *Lhx2*, the loss of Emx2 has much smaller effects and more often causes increased expression frequencies. Lhx2:Emx2 double mutants show opposing effects on *Olfr15* expression that reveal independent effects of these two transcription factors. While Lhx2 is necessary for OR expression that supports OR gene choice, Emx2 can act differently; perhaps by helping to control the availability of OR genes for expression.

## Significance Statement

The nervous system develops myriad different types of neurons, many having numerous subtypes. The olfactory epithelium takes this to an extreme, with >1000 different subtypes of the olfactory sensory neuron (OSN), each defined by the expression of a single odorant receptor (OR) gene. We demonstrate that Lhx2 is the homeodomain transcription factor necessary to drive the expression of OR genes. By stabilizing the expression of a single OR gene and consequently triggering OSN maturation, Lhx2 drives the events that complete the process of OR gene choice, thereby defining the role of each OSN in odor detection and discrimination.

## Introduction

Odorant receptors (ORs), which often number >1000 and therefore comprise the largest family of mammalian genes, are G-protein-coupled receptors that evolved to transduce the binding of odorant molecules into intracellular signals (DeMaria and Ngai, 2010). In a process called OR gene choice, each olfactory sensory neuron (OSN) selects one allele of one OR gene for high-level expression (Rodriguez, 2013). The choice is random, except that each OR gene is available for expression only in a defined region of the olfactory epithelium called a zone. This exquisite specificity is essential for odor discrimination (Fleischmann et al., 2008).

Repressive chromatin modifications characteristic of heterochromatin are fundamental to OR gene choice (Lyons and Lomvardas, 2014). These modifications require the histone methyltransferases Kmt1c and Kmt1d, appear early in the OSN cell lineage before the initiation of OR gene expression, and ultimately result in the compaction of OR genes into heterochromatin compartments in OSN nuclei (Magklara et al., 2011; Clowney et al., 2012; Lyons et al., 2014). Derepression of OR alleles occurs in immature OSNs, or perhaps their immediate progenitors, and requires the histone demethylase Kdm1a (Lyons et al., 2013). Once the expression of an OR allele initiates, OR protein accumulation in the ER triggers a feedback signal that represses *Kdm1a* so that no other OR allele can be derepressed (Dalton et al., 2013). The expression of an OR appears to trigger the final stages of OSN differentiation, resulting in a mature OSN that expresses a single OR allele.

What drives transcription from the newly derepressed OR allele is not fully understood. However, the enhancers and proximal promoters of OR genes contain two conserved elements, homeodomain sites and Olf1/Ebf1 (O/E-like) sites, whose mutation reduces OR gene expression (Bozza et al., 2002; Serizawa et al., 2003; Rothman et al., 2005; Hoppe et al., 2006; Michaloski et al., 2006; Vassalli et al., 2011; Clowney et al., 2012; Plessy et al., 2012; Markenscoff-Papadimitriou et al., 2014). O/E-like sites are bound by Ebf transcription factors, but whether they are required for OR gene expression is uncertain (Wang et al., 2004). Yeast one-hybrid experiments reveal that the homeodomain sites can be bound by several transcription factors, but Lhx2 and Emx2 are the two most strongly implicated in OR gene expression (Hirota and Mombaerts, 2004; Hoppe et al., 2006; Hirota et al., 2007; McIntyre et al., 2008). Unfortunately, germ-line deletions of these transcription factors are confounded by large reductions in OSN number. *Emx2*-null mice have 40% fewer mature OSNs, and *Lhx2*-null mice are even more strongly affected, being nearly devoid of mature OSNs and having fewer immature OSNs (Hirota and Mombaerts, 2004; Kolterud et al., 2004).

Lhx2 is a LIM (Lin-11, Isl-1, Mec-3) homeodomain transcription factor that is important for the development of diverse tissues. In the nervous system, it tends to be critical for neural versus glial specification. For example, it regulates progenitor cell fate and proliferation in the cerebral cortex, hypothalamus, and retina, often by controlling the expression of transcription factors and signaling proteins (Chou and O'Leary, 2013; Gordon et al., 2013; Shetty et al., 2013; Roy et al., 2014; Hsu et al., 2015). Lhx2 also acts later in cell differentiation, supporting the final differentiation of tanycytes in the hippocampus and Müller glia in the retina (Salvatierra et al., 2014; de Melo et al., 2016). Emx2 (empty spiracles homeobox 2) has similar roles in neural development. It contributes to the development of portions of the telencephalon, where it regulates cell specification and neural circuit formation (Pellegrini et al., 1996; Fukuchi-Shimogori and Grove, 2003; Hamasaki et al., 2004; O'Leary et al., 2007; Dwyer et al., 2011).

To test the effects of the loss of Lhx2 and Emx2 on OR expression, we bred mice that allow conditional deletion of *Lhx2* and *Emx2* in OSNs, finding that Lhx2 is necessary for OR gene expression and consequent differentiation of OSNs. Emx2, in contrast, appears to be necessary for OR gene expression in a distinctly different way.

## Materials and Methods

### Mice

123Cre transgenic mice and OmpCre gene-targeted mutant mice, respectively, were the gifts of Dr. Y. Yoshihara (RIKEN Brain Science Institute, Wako, Japan) and Dr. P. Mombaerts (Max Planck Research Unit for Neurogenetics, Frankfurt am Main, Germany). Lhx2^fl/+^ mice and Emx2^fl/+^ mice were the gifts of Dr. D. O’Leary (Salk Institute for Biological Studies, La Jolla, CA). Both sexes were used in all experiments, and no mouse carried more than one copy of Cre recombinase.

Z/EG reporter mice (Novak et al., 2000) were purchased from The Jackson Laboratory (https://www.jax.org/strain/003920).

All treatments and procedures used with mice were approved by the university institutional animal care and use committee and were consistent with National Institutes of Health guidelines on animal use in research.

### DNA microarray

Olfactory mucosae were dissected from mice of the desired age and genotype. Total RNA was prepared using TriReagent as directed by the manufacturer (Molecular Resource Center).

Given that ORs are detectably expressed by age postnatal day 1 (P1; Rodriguez-Gil et al., 2009) to best match previous work on germ-line-targeted deletion of *Emx2* (McIntyre et al., 2008) experiments used age P1 neonatal Emx2^fl/+^ mice. Conditional deletion of *Lhx2* in immature OSNs was also assessed in neonates at age P3. Conditional deletion of *Lhx2* in mature OSNs and the double deletion of *Lhx2* and *Emx2* were assessed in juvenile mice at age P26. Each of the two or three litters from which conditional knock-out mice were obtained for an experiment also contributed control mice. Preliminary testing showed that OR expression in 123Cre:Lhx2^fl/+^ mice did not differ from Lhx2^fl/+^ mice, arguing that the deletion of a single *Lhx2* allele has no effect. Littermate controls therefore were any genotype where no more than one allele could undergo recombination.

Preparation of samples for microarray hybridization and initial data reduction were performed as described previously by the University of Kentucky Microarray Core Facility, using equal amounts of total RNA from each sample (McIntyre et al., 2008). GeneChip Mouse Gene 1.0 ST arrays were used to quantify mRNAs. Data were analyzed at the transcript cluster level, which combines the signals from the probe sets representing all exons of each gene into a single value. This GeneChip has 1142 clusters of exon-level probe sets, called transcript clusters, which represent 1098 functional OR genes (Table 1). Given that the vast majority of OR genes are represented unambiguously by a transcript cluster, we use the terms OR gene or OR mRNA in reporting and discussing these data. Trace amine-associated receptor (TAAR) mRNA data from the GeneChip were also analyzed. Except for *Taar8a*, each TAAR gene is represented by at least one unambiguous transcript cluster. In mice, *Taar7c* is a pseudogene.

**Table 1.** GeneChip Mouse Gene 1.0 ST array probe set OR transcript clusters

Type	Transcript clusters (*n*)	OR genes (*n*)
Single functional gene	996	994
Single pseudogene	34	34
Multiple gene	112	104
No functional gene	2	

To normalize to the number of mature OSNs present in the samples of olfactory mucosae used in these experiments, we took advantage of the recent identification of hundreds of mature and immature OSN-specific mRNAs (Nickell et al., 2012). There are 581 mature OSN-specific mRNAs and 697 immature OSN-specific mRNAs that are consistently detected by Affymetrix GeneChip Mouse Gene 1.0 ST arrays. The average difference in the abundance of the 581 mature OSN-specific mRNAs between mutants and littermate controls was used as a normalization factor. Statistical analysis of the data was performed via *t* tests. Correction for multiple testing was performed using a stepwise false discovery rate (FDR) procedure (Benjamini and Hochberg, 1995; Storey and Tibshirani, 2003a,b). The microarray data have been deposited at Gene Expression Omnibus (superseries record GSE74527, subseries GSE74522–GSE74526).

### *In situ* hybridization and immunofluorescence

The *in situ* hybridization methods that Ishii et al. (2003, 2004) have described in detail were followed using 10–12 µm coronal cryosections from mice in the age range P21–P34. We have previously described the use of these procedures to locate cells expressing >300 mRNAs in olfactory epithelia (Sammeta et al., 2007; Nickell et al., 2012; Heron et al., 2013). cDNA fragments of each mRNA were amplified by PCR from olfactory mucosal cDNA and cloned into pBluescript (Table 2). The fragments chosen were selected to have <90% identity with any other mouse mRNA. Recombinant RNA probes labeled with digoxigenin were prepared for each mRNA species. Sense controls were invariably negative.

**Table 2.** *In situ* hybridization probe cDNAs

Gene symbol	NCBI accession no.	Region used
Omp	NM_011010	569-1064
Gap43	NM_008083	369-835
Lhx2	NM_001290646	532-1251
Olfr6	NM_206897	237-741
Olfr15	NM_008762	172-658; 477-932
Olfr17	NM_020598	208-684
Olfr90	NM_146477	371-866
Olfr129	NM_146327	692-1139
Olfr156	NM_019474	125-598
Olfr308	NM_146621	25-882
Olfr545	NM_146840	1-827
Olfr615	NM_147080	1-942
Olfr963	NM_001011827	138-851
Olfr1440	NM_146684	229-726
Olfr1465	NM_001011841	35-454

Probes from the two fragments of Olfr15 give identical results. The region of Lhx2 used is contained within exons 1–3, the floxed region of the mutated *Lhx2* gene.

Immunohistochemistry was also performed on coronal cryosections from the tissue of age P31–P34 mice, prepared as described above except that fixation in 4% paraformaldehyde was performed for only 60–90 min rather than overnight. These frozen sections were thawed and permeabilized in PBS, pH 7.4, with 1% Triton X-100. Blocking buffer (2% BSA and 0.4% Triton X-100 in PBS) was applied for 1 h at room temperature followed by incubation in primary antibody diluted in the blocking buffer overnight at 4˚C. Sections were washed with 0.05% Tween-20 in PBS for 30 min and then incubated with Cy3-conjugated secondary antibody diluted in PBS. Nuclei were counterstained using Hoechst 33342 (catalog #H1399, Invitrogen). The anti-Casp3 rabbit polyclonal antibody specifically detects a 17 kDa cleavage fragment produced by apoptosis-initiating proteases. The specificity of this antibody is well documented (Hu et al., 2000; Cheong et al., 2003; Kaiser et al., 2008). Phosphorylated histone 3, which increases abruptly as chromatin condenses during mitosis, was detected using a rabbit polyclonal antibody (catalog #06-570, Millipore) at a 1:200 dilution. The specificity of this antibody for its intended target has long been known (Mauser et al., 2002). Rabbit anti-Gap43 (AB5220, Millipore) was used at a 1:200 dilution to identify immature OSNs and goat anti-olfactory marker protein (OMP; catalog 544-10001, Wako) was used at a dilution of 1:1000 to identify mature OSNs. The specificity of these two antibodies for their intended targets is widely recognized (Baker et al., 1989; Song et al., 2002; Inaki et al., 2004; Koo et al., 2005; McIntyre et al., 2010; Miller et al., 2010). CD68 was detected using a rat antibody (clone FA-11, catalog #MCA1957, AbD Serotec) at a dilution of 1:500 in a different blocking buffer, as follows: 2% normal donkey serum, 0.03% Triton X-100 in PBS. The specificity of this antibody for CD68 is widely accepted and has been confirmed by targeted gene deletion of CD68 (Rabinowitz and Gordon, 1991; Song et al., 2011).

For secondary antibodies, a Cy3-conjugated donkey anti-rabbit IgG (catalog #711-165-152, Jackson ImmunoResearch) and a Cy3-conjugated donkey anti-goat IgG (catalog #705-165-147, Jackson ImmunoResearch) were used at a dilution of 1:1000 in blocking buffer. A Cy3-conjugated donkey anti-rat IgG (catalog #712-165-150, Jackson ImmunoResearch) was used at 1:500 in 2% normal donkey serum and 0.03% Triton X-100 in PBS.

Wide-field images of labeling in tissue sections were obtained on a Nikon Diaphot 300 inverted microscope using a Spot 2e camera and Spot software version 4.0.6 through a 40×/0.75 numerical aperture Plan Fluor objective, a 20×/0.75 numerical aperture Plan Apo differential interference contrast (DIC) objective, a 10×/0.45 numerical aperture Plan Apo DIC objective, or a 4×/0.13 numerical aperture Plan objective. Images were processed in Adobe Photoshop only by adjusting size and brightness. To produce final figures, images were combined and labeled in Canvas Draw version 2.0 (ACD Systems).

For counts of labeled cells, coronal sections matched for anterior–posterior position between mutant and control were selected and used to generate an average count per linear distance along the olfactory epithelium for each mouse. The zonal expression patterns of ORs presents a special problem when normalizing the number of OSNs expressing an OR to the amount of olfactory epithelium assessed because the boundaries of OR expression zones are difficult to locate. We therefore chose a conservative approach, including only the distance between the outermost OSNs expressing the targeted OR. This overestimates the frequency of labeled OSNs, and therefore favors making a type II error over a type I error. In cases where OR genes are too rarely expressed to use this strategy, we measured the number of labeled OSNs per tissue section.

Cell counts of OR expression were normalized to the fractional loss of mature OSNs in the mutant mice, as measured by *in situ* hybridization for Omp mRNA from adjoining sections of the same mice. The average difference from control genotypes was used to calculate a correction factor that was applied to the counts.

For consistency in the immunohistochemistry data, counts of immunoreactive cells were made along the septum. To restrict the measure of mitosis to progenitors of OSNs, only cells immunoreactive for phosphorylated histone 3 in the basal layer of the olfactory epithelium were counted. As OSNs are the only cells in which genes were recombined in this project, cells immunoreactive for the active fragment of Casp3 were counted in the OSN layers of the olfactory epithelium. In contrast, all CD68-immunoreactive cells within the olfactory epithelium were counted. Correction for multiple testing within the cell count data was performed using a stepwise FDR procedure (Benjamini and Hochberg, 1995; Storey and Tibshirani, 2003a,b). The average values calculated for these cell counts are reported with their SDs.

In fluorescent images from sections of Z/EG reporter mice, nuclei inside GFP^+^ and GFP^−^ OSN cell bodies were counted per linear distance along the olfactory epithelium.

## Results

### Conditional *Lhx2* deletion in immature OSNs decreases OR expression frequency

In addition to expression in basal cells of the olfactory epithelium, Lhx2 is present in OSNs, the cells that express OR genes (Hirota and Mombaerts, 2004; Kolterud et al., 2004). Lhx2 interacts directly with OR gene promoters in yeast one-hybrid assays and binds OR gene enhancers *in vivo* (Hoppe et al., 2003; Hirota and Mombaerts, 2004; Markenscoff-Papadimitriou et al., 2014). These data predict that Lhx2 plays a direct and positive role in OR gene expression.

**Figure 1.**
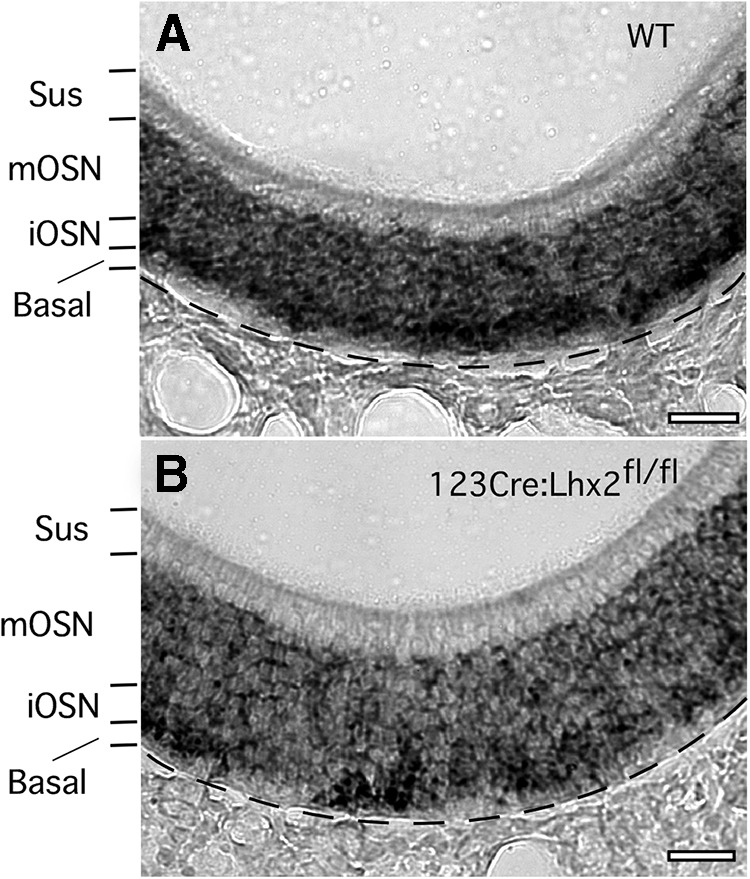
Most OSNs in 123Cre:Lhx2^fl/fl^ mice continue to express Lhx2. ***A***, *In situ* hybridization for Lhx2 mRNA in a wild type (WT) mouse. ***B***, *In situ* hybridization for Lhx2 mRNA in a 123Cre:Lhx2^fl/fl^ mouse. Sus, Sustentacular cell layer; mOSN, mature OSN cell layer; iOSN, immature OSN cell layer; Basal, basal cell layer. Scale bars, 40 µm.

**Figure 2.**
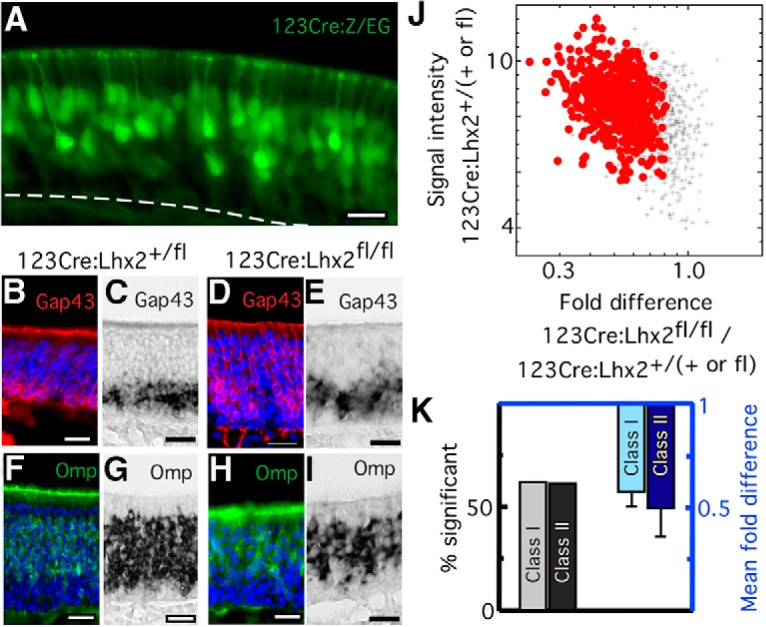
Conditional deletion of *Lhx2* in immature OSNs decreases the abundance of most OR mRNAs. ***A***, In 123Cre:Z/EG mice, recombination-dependent GFP expression marks OSNs in the immature and mature OSN layers of the olfactory epithelium. Dashed line, location of the basal lamina separating the olfactory epithelium and lamina propria. Scale bar, 20 µm. ***B–E***, Gap43 immunoreactivity (***B***, ***D***) and *in situ* hybridization for Gap43 mRNA (***C***, ***E***) identify immature OSN cell bodies in 123Cre:Lhx2^fl/fl^ mice (***D***, ***E***) and littermate controls (***B, C***). ***F–I***, Omp immunoreactivity (***F***, ***H***) and *in situ* hybridization for Omp mRNA (***G***, ***I***) identify mature OSN cell bodies in 123Cre:Lhx2^fl/fl^ mice (***H***, ***I***) and littermate controls (***F***, ***G***). Scale bars, 20 µm. ***J***, In 123Cre:Lhx2^fl/fl^ mice, the abundance of 676 OR mRNAs decreases (*p* < 0.01; *n* = 4; FDR <1%). •, Significant decrease; +, no difference from littermate controls. ***K***, The percentage of class I ORs affected by *Lhx2* deletion in immature OSNs is very similar to the percentage of Class II ORs affected, and the magnitude of the decreases are similar for both classes of ORs. Error bars represent SDs.

To try to minimize the loss of OSNs that would be expected if OR expression fails, and that confounds the interpretation of data from germ-line deletion of *Lhx2* (Hirota and Mombaerts, 2004; Kolterud et al., 2004; Hirota et al., 2007), we turned to a conditional deletion strategy. We obtained 123Cre mice in which Cre expression begins in immature OSNs (Takeuchi et al., 2010). We produced 123Cre:Lhx2^fl/fl^ mice by crossing 123Cre mice with floxed Lhx2 mice in which LoxP sites bound the first three exons of *Lhx2* (Chou et al., 2009). Using *in situ* hybridization with a probe sequence contained within these three floxed exons, we find that the deletion of *Lhx2* is incomplete in these mice (Fig. 1). To confirm that Cre recombinase driven by the 123 promoter is effective, we crossed 123Cre mice with Z/EG mice. GFP expression reports Cre-mediated recombination in 48 ± 1% (*n* = 2 mice) of the cell bodies in the OSN layers of these mice (Fig. 2*A*). All of the fluorescent cells show the morphology of neurons. No fluorescence is present in other cells or cell processes found in the OSN layers, such as infiltrating macrophages, the basal processes of sustentacular cells, and the ducts of Bowman’s glands. On average, the basal end of the GFP fluorescent cell body layer is 16.2 ± 0.8% of the distance from the basement membrane to the apical surface in these mice, which is consistent with recombination in immature OSNs. Given that complete deletion of *Lhx2* might result in the loss of most mature OSNs and thereby confound further experiments, we reasoned that incomplete deletion could provide a situation that allows the effects on OR expression to exceed the loss of OSNs.

In 123Cre:Lhx2^fl/fl^ mice, the numbers of Gap43^+^ immature OSNs are identical to those of control mice (Fig. *2B–E*), and the average abundance of 697 immature OSN-specific mRNAs (Nickell et al., 2012) is similarly unaffected. The number of mature OSNs is slightly reduced in 123Cre:Lhx2^fl/fl^ mice (Fig. 2*F–I*). The average abundance of 581 mature OSN-specific mRNAs (Nickell et al., 2012) is 86 ± 14% that of controls (*n* = 4 mice), and the thickness of the layer of Omp^+^ OSN cell bodies after *in situ* hybridization is 82% that of controls on average. This small decrease indicates a difference in the survival or production of mature OSNs compared with control littermates.

When assessing the abundance of OR mRNAs, the effect of a small decrease in mature OSN number can be eliminated by simple normalization to the extant fraction of mature OSNs as long as accelerated OSN turnover is not responsible for the decrease in mature OSNs. To test the turnover of OSNs, we measured the frequency of apoptosis in the OSN layers of the olfactory epithelium by active Casp3 immunoreactivity. 123Cre:Lhx2^fl/fl^ mice show no difference from littermate controls in this measure of apoptosis (*p* = 0.5627, *n* = 3 mice), demonstrating that OSN turnover is near normal levels in these mice. The measures of OR mRNA abundance can therefore be safely adjusted by normalization to the fraction of mature OSNs present.

In 123Cre:Lhx2^fl/fl^ mice, the predominant effects on mRNA abundance are on OR mRNAs, which is reminiscent of our assessment of *Emx2* germ-line mutants (McIntyre et al., 2008). The significantly affected mRNAs are mostly ORs, and OR mRNAs show larger effects compared with littermate controls than other types of transcripts. Even after correction for the 14% decrease in mature OSNs, 676 OR mRNAs are significantly less abundant in 123Cre:Lhx2^fl/fl^ mice than in control mice (Fig. 2*J*; *p* < 0.01; FDR <1%). Implicit in the finding of significant differences in the normalized data, but worth noting explicitly, is that the 14% loss of mature OSNs is incapable of accounting for the decreased abundance of OR mRNAs. The average reduction for all OR mRNAs is 41% after normalization. For the 676 OR mRNAs with a significant decrease, the average reduction is 50%, arguing that a fraction of OSNs must be experiencing altered OR expression.

Data from germ-line *Lhx2* knockouts suggested that Lhx2 might act specifically on the class II OR genes, which arose during mammalian evolution (Hirota et al., 2007). However, in 123Cre:Lhx2^fl/fl^ mice the affected mRNAs include both the more evolutionarily ancient class I ORs, which are recognizably similar to the ORs of fishes, and the larger, more diverse group of class II ORs (Fig. 2*K*). Class I and class II OR mRNAs show similar reductions in quantity, and similar fractions of ORs are significantly affected.

Some OSNs use TAARs, rather than ORs, to detect odor molecules. Like ORs, TAARs are G-protein-coupled receptors. All but *Taar1* of the 14 functional TAAR genes in mice appear to be expressed in OSNs, where they act as receptors for amine-containing odorants (Liberles and Buck, 2006; Johnson et al., 2012). Six TAAR mRNAs (Taar7d,e,f, Taar8b,c, and Taar9) are significantly reduced in 123Cre:Lhx2^fl/fl^ mice.

The large effects on OR mRNA abundance derive from relatively small effects on Lhx2 mRNA abundance. In samples of olfactory mucosae from 123Cre:Lhx2^fl/fl^ mice, Lhx2 mRNA is decreased by only 15% (*p* = 0.0952, *n* = 4). This small reduction in Lhx2 mRNA abundance is consistent with the data shown in Figure 1, and Figure 2*A* similarly predicts that the recombination of both *Lhx2* alleles occurs in only a fraction of immature OSNs.

**Figure 3.**
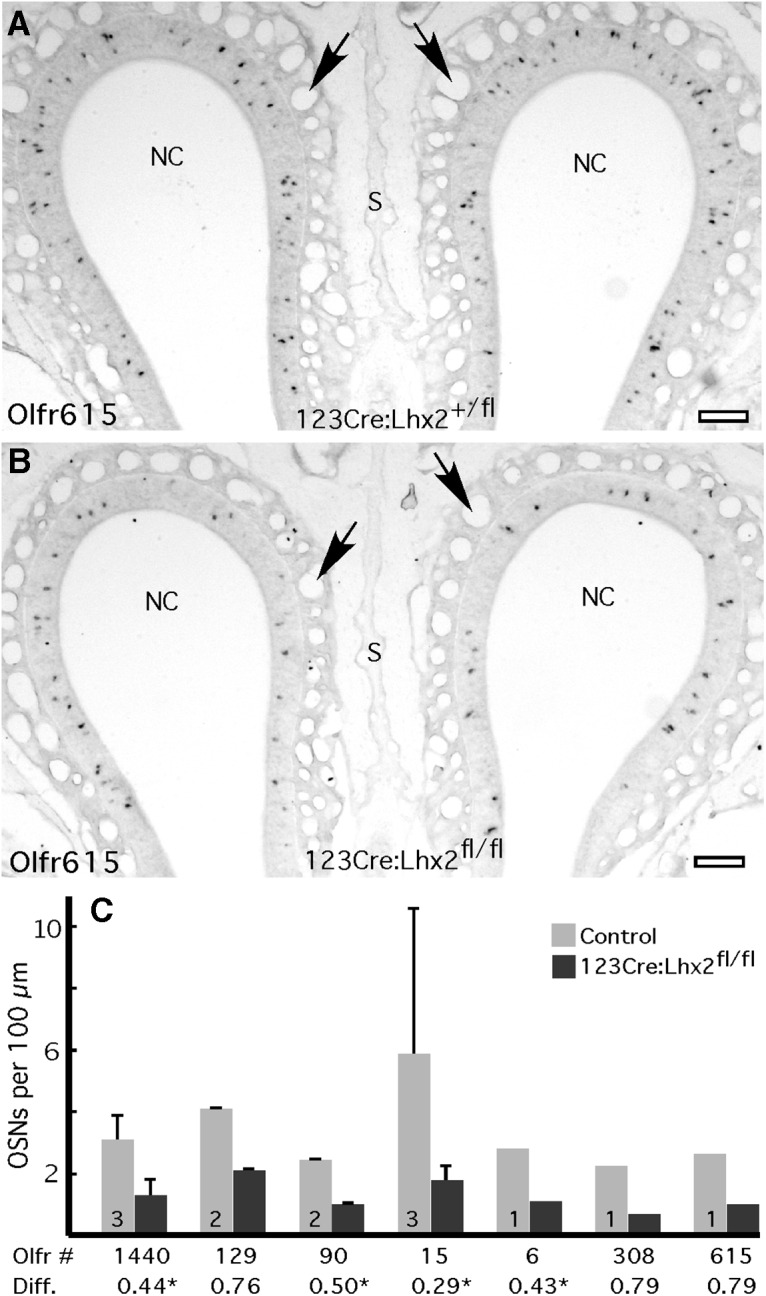
Conditional deletion of *Lhx2* in immature OSNs reduces OR gene expression frequencies. ***A***, *In situ* hybridization for *Olfr615* labels scattered OSNs in the dorsal olfactory epithelium. ***B***, In 123Cre:Lhx2^fl/fl^ mice, the frequency of OSNs expressing *Olfr615* appears to be reduced. ***C***, Normalized cell counts show that ORs are consistently less frequently expressed in 123Cre:Lhx2^fl/fl^ mice compared with littermate controls. Numbers in control bars are the numbers of littermate pairs tested. Diff., Normalized fold differences from the microarray experiment; *Significant difference (*p* < 0.01; *n* = 4; FDR <1%). NC, Air space of the nasal cavity; S, nasal septum; arrows, blood vessels in the lamina propria. Scale bars, 100 µm.

The decreased abundance of OR mRNAs arises from reduced frequencies of OR expression rather than from reduced amounts of OR mRNA per OSN. For OR mRNAs that decrease in abundance, *in situ* hybridization consistently reveals fewer OSNs expressing each OR tested. Figure 3 shows an example of a moderately affected mRNA, Olfr615, along with quantitative data on differences in expression frequencies for seven ORs. Even OR genes that do not reach our conservative criteria for significance in the expression profiling experiment, such as *Olfr308*, *Olfr129*, and *Olfr615*, consistently show lower frequencies of expression. The OR mRNA in each OSN is one of the most abundant transcripts in the cell; thus, the latency to the appearance of the signal for OR mRNAs during *in situ* hybridization (<24 h in our hands) is shorter than that of most mRNAs. Allowing the reaction to develop for 48 h in conditional deletion mice does not increase the frequency of labeled OSNs.

One way for the loss of Lhx2 to cause changes in OR mRNA abundance and OR expression frequency is to disrupt the patterns of zonal expression of OR genes. However, the *in situ* hybridization data reveal that reduced frequencies of OR expression are not accompanied by changes in the zonal patterns of OR expression in 123Cre:Lhx2^fl/fl^ mice. Each of the seven OR mRNAs tested in 123Cre:Lhx2^fl/fl^ mice is transcribed from a gene that continues to be expressed in the region of the olfactory epithelium in which the OR is normally expressed, albeit less frequently. Altered zonal expression patterns are not responsible for the effects that we observe.

### *Lhx2* deletion in mature OSNs decreases OR expression frequency

The data from 123Cre:Lhx2^fl/fl^ mice confirm that Lhx2 has a positive effect on OR expression and is needed for the development of normal OR expression frequencies. However, once OSNs select a single OR allele for expression and become mature OSNs, the repressive epigenetic modification of OR gene loci might be immutable, permanently preventing the expression of any other OR genes. If this is true—and if most mature OSNs survive the loss of OR expression—the deletion of *Lhx2* after OSNs reach maturity should have little effect on OR expression frequencies, but might decrease the amount of OR mRNA per OSN. Alternatively, if the feedback mechanisms that prevent the expression of additional OR genes must be continuously active in order to maintain the singularity of the OR gene choice, or if OR expression simply ceases, the loss of Lhx2 in mature OSNs could result in altered OR expression frequencies.

**Figure 4.**
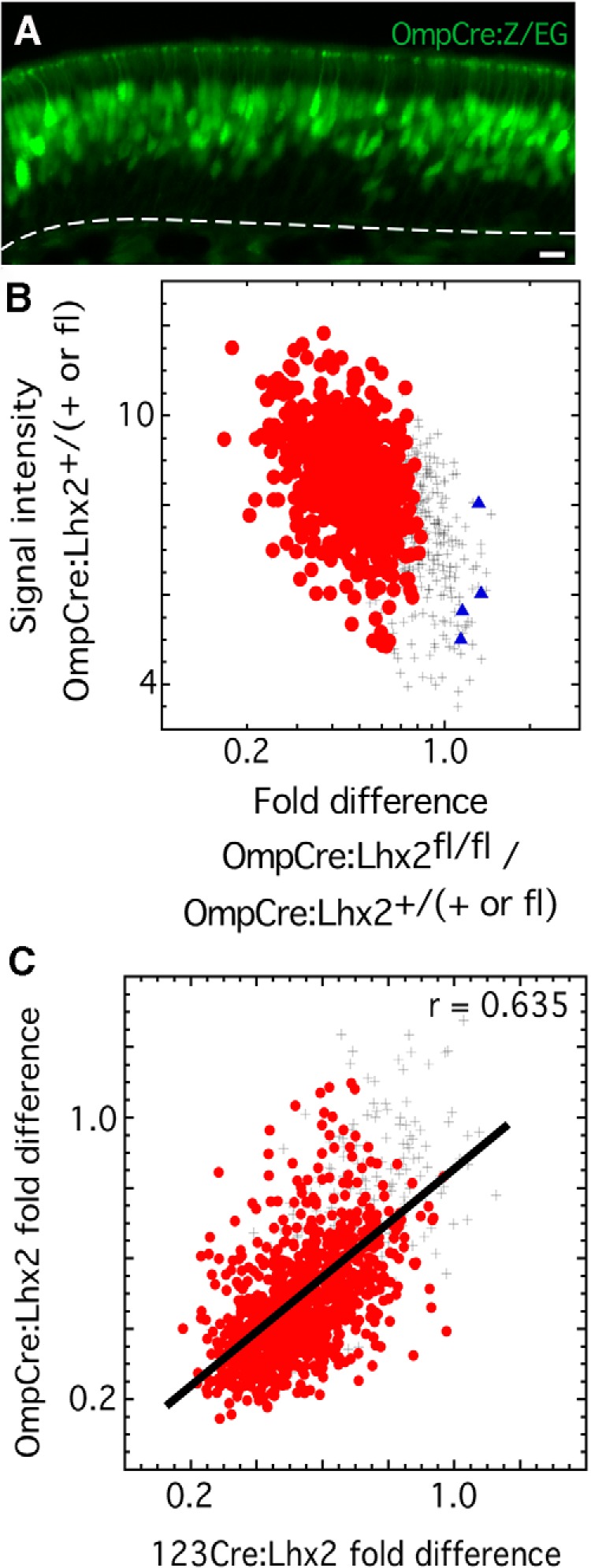
Deletion of *Lhx2* in mature OSNs decreases the abundance of most OR mRNAs. ***A***, In OmpCre:Z/EG mice, recombination-dependent GFP expression marks OSNs in the mature OSN layer. Dashed line, Location of the basal lamina separating the olfactory epithelium and lamina propria. Scale bar, 20 µm. ***B***, In OmpCre:Lhx2^fl/fl^ mice, the abundance of 765 OR mRNAs is decreased relative to littermate controls (*p* < 0.01; *n* = 4; FDR <1%). •, Significant decrease; +, no difference from littermate controls; ▴, increased abundance. ***C***, Deletion of *Lhx2* in immature OSNs (123Cre:Lhx2) and mature OSNs (OmpCre:Lhx2) produced similar effects on the abundance of most OR mRNAs relative to littermate controls. Line, Pearson linear correlation fit; •, significant decrease in at least one of the two datasets; +, no decrease compared with littermate controls.

To drive the expression of Cre recombinase specifically in mature OSNs, we used Omp-Cre mice (Li et al., 2004). These mice have a targeted insertion of *Cre* into the *Omp* gene locus, which is expressed only in mature OSNs in the olfactory epithelium and rarely expressed in other tissues (Farbman and Margolis, 1980; Monti Graziadei, 1983). In Omp-Cre mice carrying the Z/EG reporter transgene, 41 ± 1% (*n* = 3 mice) of cells in the mature OSN layer of the epithelium show GFP fluorescence (Fig. 4*A*), demonstrating Cre recombinase activity in mature OSNs. On average, the basal end of the GFP fluorescence layer is 34.4 ± 3.6% of the distance from the basement membrane to the apical surface, which is consistent with labeling restricted to mature OSNs.

As in 123Cre:Lhx2^fl/fl^ mice, recombination of *Lhx2* is successful in OmpCre:Lhx2^fl/fl^ mice and causes only small changes in the number of OSNs. The average decrease in Lhx2 mRNA in samples of olfactory mucosae from OmpCre:Lhx2^fl/fl^ mice is 16% (*p* = 0.002613, *n* = 4). The average abundance of 697 immature OSN-specific mRNAs in OmpCre:Lhx2^fl/fl^ mice is 1.06 ± 0.13-fold that of littermate controls, indicating that the numbers of immature OSNs are normal or perhaps slightly elevated. The average abundance of 581 mature OSN-specific mRNAs is reduced 12% on average compared with littermate controls, indicating a small reduction in the number of mature OSNs. Just as in 123Cre:Lhx2^fl/fl^ mice, OmpCre:Lhx2^fl/fl^ mice show no evidence of increased OSN turnover. We find no difference in the frequency of active Casp3-immunoreactive cells in the olfactory epithelia of these mice (*p* = 0.2473, *n* = 3 mice).

Even though Lhx2 is not lost until OSNs reach maturity in OmpCre:Lhx2^fl/fl^ mice, the effects on OR and TAAR mRNAs are as widespread as in 123Cre:Lhx2^fl/fl^ mice (Fig. 4*B*). After normalizing to 12% fewer mature OSNs, we detect decreased abundance of 765 OR mRNAs and 12 TAAR mRNAs (*p* < 0.01; *n* = 4; FDR < 1%). The significantly reduced TAAR mRNAs are Taar2, Taar3, Taar4, Taar5, Taar6, Taar7a, 7b, 7d, 7e, 7f, Taar8a, and Taar9. In addition, four OR mRNAs show significant increases in abundance. In order of decreasing fold difference, they are as follows: Olfr1465, Olfr401, Olfr574, and Olfr1462.

The similarity of effects on OR mRNA abundance in OmpCre:Lhx2^fl/fl^ mice and 123Cre:Lhx2^fl/fl^ mice suggests that Lhx2 is acting consistently on OR gene control elements at both stages of OSN differentiation. If so, the same ORs should be affected in both conditional deletion strains. This proves correct, with 530 OR mRNAs showing significant decreases in both 123Cre:Lhx2^fl/fl^ mice and OmpCre:Lhx2^fl/fl^ mice. Not only are the same ORs affected, the majority of ORs tend to show similar fold differences in abundance in OmpCre:Lhx2^fl/fl^ mice and 123Cre:Lhx2^fl/fl^ mice (Fig. 4*C*). The average reduction in OR mRNA abundance in OmpCre:Lhx2^fl/fl^ mice is 41% after normalization. For ORs that reach significance, the average reduction is 53%.

**Figure 5.**
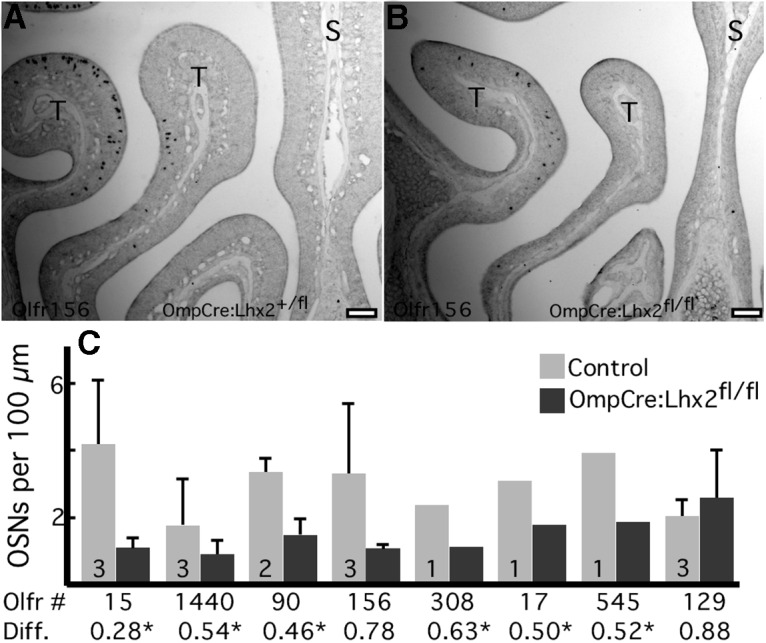
Conditional deletion of *Lhx2* in mature OSNs reduces OR expression frequencies. ***A***, *In situ* hybridization shows that *Olfr156* is expressed in scattered OSNs in the ventral olfactory epithelium. ***B***, In OmpCre:Lhx2^fl/fl^ mice, the frequency of OSNs expressing *Olfr156* is reduced. ***C***, Normalized cell counts from *in situ* hybridization images show that the ORs tested are less frequently expressed in OmpCre:Lhx2^fl/fl^ mice compared with littermate controls, which is in agreement with normalized fold differences (Diff.) measured in the microarray experiment. *Significant difference (*p* < 0.01; *n* = 4; FDR < 1%). T, Turbinate; S, nasal septum. Scale bars, 100 µm.

Just as in 123Cre:Lhx2^fl/fl^ mice, the decreased abundance of OR mRNAs in OmpCre:Lhx2^fl/fl^ mice is associated with decreased frequencies of expression of these ORs (Fig. 5), an effect that is not due to increased latencies for signal development during *in situ* hybridization. The four OR mRNAs that increase in abundance are more difficult to assess because they have very low expression frequencies. For example, *in situ* hybridization for Olfr1465, which shows the largest increase at 1.3-fold, detects fewer than one labeled cell per tissue section. Counts of labeled cells reveal no difference between *Lhx2* conditional deletion mice and controls (*p* = 0.3152; *n* = 3 mice), with OmpCre:Lhx2^fl/fl^ mice at 0.42 ± 0.38 cells per tissue section and littermate controls at 0.66 ± 0.70 cells per tissue section.

### *Emx2* deletion in OSNs has small effects on OR expression

OR expression is not completely lost after conditional deletion of *Lhx2* in OSNs. Incomplete deletion of *Lhx2* across the population of OSNs must be responsible for much of the continued expression of ORs, but contributions from other mechanisms are also possible. For example, other transcription factors might substitute for Lhx2 or may be sufficient in some cases to drive OR gene transcription by themselves. Among homeodomain transcription factors that could substitute for Lhx2, the most promising candidate is Emx2. Like Lhx2, Emx2 was captured in a yeast one-hybrid assay using an OR promoter sequence (Hirota and Mombaerts, 2004). Embryonic mice with a germ-line deletion of *Emx2* show altered abundance of many OR mRNAs due to altered expression frequencies (McIntyre et al., 2008). Reassessing these data using the normalization procedure applied to our conditional *Lhx2* mutant data, 159 OR mRNAs are significantly decreased, and 52 are increased in the germ-line *Emx2* deletion (*p* < 0.05; FDR < 10%). We tested whether delaying the deletion of *Emx2* until the OSN cell lineage reaches the OSN stages would have similar effects by breeding 123Cre:Emx2^fl/fl^ mice and OmpCre:Emx2^fl/fl^ mice.

In both strains, the *Emx2* gene locus is successfully recombined. In 123Cre:Emx2^fl/fl^ mice, Emx2 mRNA is reduced by 48% (*p* = 0.00001; *n* = 4). In OmpCre:Emx2^fl/fl^ mice, Emx2 mRNA is decreased 32% (*p* = 0.00059; *n* = 4). Lhx2 mRNA abundance is not affected in either 123Cre:Emx2^fl/fl^ mice (fold difference, 0.99, *p* = 0.9343; *n* = 4) or OmpCre:Emx2^fl/fl^ mice (fold difference, 1.03, *p* = 0.6205; *n* = 4). Unlike germ-line deletion of *Emx2*, the numbers of mature and immature OSNs are unaffected after conditional *Emx2* deletion.

OR mRNA abundance is only mildly affected by conditional deletion of *Emx2* in OSNs. Only 34 OR mRNAs show *p* < 0.01 in these experiments (Table 3), and none of these have an FDR of <1%. In 123Cre:Emx2^fl/fl^ mice, 6 of these 34 OR mRNAs increase in abundance, but these same mRNAs are not significantly more abundant in OmpCre:Emx2^fl/fl^ mice. The values in Table 3 suggest that the magnitude of the effects of Emx2 deletion differ between the two strains, a hypothesis that proves true. The average absolute value of the fold difference in 123Cre:Emx2^fl/fl^ mice compared with littermate controls is 0.34 ± 0.21, compared with 0.21 ± 0.12 for OmpCre:Emx2^fl/fl^ mice (*p* = 0.0064, *n* = 4; two-tailed paired *t* test). The ability of Emx2 to impact OR expression appears to decrease with OSN maturation.

**Table 3.** OR mRNAs significantly affected in conditional *Emx2* deletion mutants

Gene symbol	123Cre:Emx2 fold difference	OmpCre:Emx2 fold difference
*Olfr1087*	0.5583*	0.7984
*Olfr11*	0.7444	0.7893*
*Olfr111*	0.6708*	0.7826
*Olfr12*	1.0522	0.8621*
*Olfr1249*	0.7437	0.7533*
*Olfr1277*	1.1381	0.6814*
*Olfr130*	0.9735	0.8090*
*Olfr1309*	0.9657	0.7151*
*Olfr1325*	0.4873	0.6895*
*Olfr1355*	0.6195*	0.7902
*Olfr1359*	0.5582	0.4949*
*Olfr1364*	0.7881	0.7082*
*Olfr1419*	0.6804*	0.7846*
*Olfr1424*	0.5759*	0.6292*
*Olfr1425*	0.8455	0.7678
*Olfr144*	1.8484*	0.9650
*Olfr1457*	0.8257	0.7559*
*Olfr1495*	0.6704*	1.0229
*Olfr15*	1.6782*	1.1469
*Olfr214*	1.7787*	0.8971
*Olfr222*	0.6071*	0.6777*
*Olfr270*	0.4249*	0.8188
*Olfr283*	0.8051*	0.8693
*Olfr291*	0.6210*	0.8153
*Olfr298*	0.3893	0.5313*
*Olfr335*	1.1136*	1.0004
*Olfr361*	0.7965	0.7296*
*Olfr42*	0.8677	0.5843*
*Olfr523*	0.8668*	0.8304
*Olfr71*	0.7472	0.8246*
*Olfr801*	0.4410*	0.8148
*Olfr90*	1.6394*	1.0124
*Olfr91*	1.3067*	0.8880
*Olfr99*	0.6356*	0.8504

* Significant difference.

Only 3 of these 34 OR mRNAs are significantly decreased in both conditional *Emx2* deletion strains. In addition, most of these 34 ORs are not specifically dependent on Emx2. Only six of them are not significantly decreased after the deletion of *Lhx2* in either immature or mature OSNs (Olfr11, Olfr71, Olfr111, Olfr270, Olfr1249, and Olfr1457).

### Additive effect of conditional deletion of both *Lhx2* and *Emx2*

Given that OR gene expression is sensitive to Lhx2 and to Emx2, the simultaneous loss of both might reveal epistasis or compensatory effects. To test this hypothesis, we generated 123Cre:Lhx2^fl/fl^:Emx2^fl/fl^ mice.

In 123Cre:Lhx2^fl/fl^:Emx2^fl/fl^ mice, Emx2 mRNA is decreased by 57% (*p* = 0.0005; *n* = 4) and Lhx2 mRNA is decreased by 27% (*p* = 0.0005; *n* = 4). Immature OSN-specific mRNAs show a small increase on average, a fold difference of 1.11 ± 0.20% (*n* = 4 mice), arguing that the immature OSN number may be slightly increased. Mature OSN-specific mRNAs show a small decrease, an average fold difference of 74 ± 25% (*n* = 4 mice). Just as with the other conditional deletion strains tested, this decrease in the number of mature OSNs is not associated with detectably increased OSN turnover. The frequency of active Casp3-immunoreactive cells in the olfactory epithelia of 123Cre:Lhx2^fl/fl^:Emx2^fl/fl^ mice is not different from that of littermate controls (*p* = 0.5271; *n* = 3).

**Figure 6.**
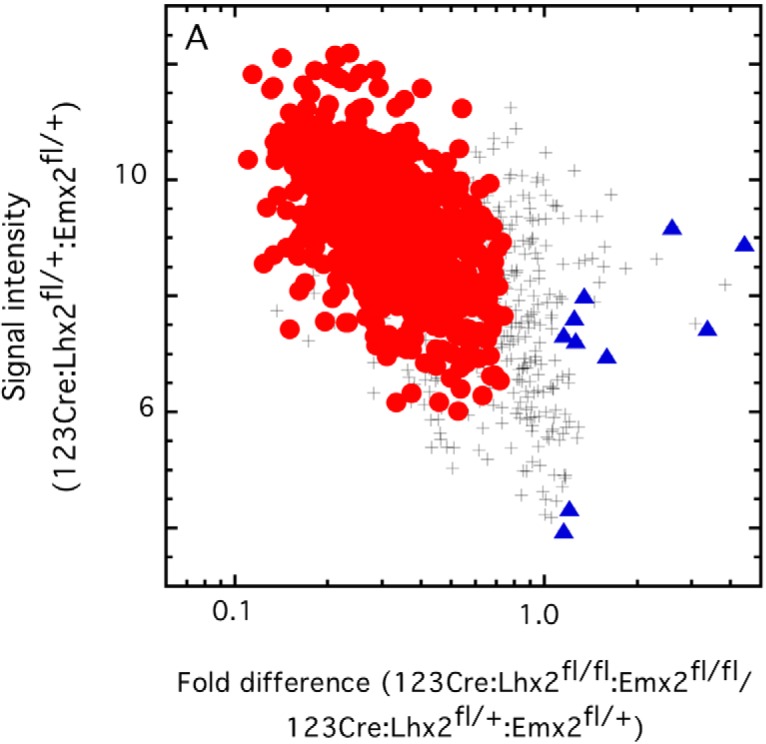
Conditional deletion of both *Lhx2* and *Emx2* in immature OSNs decreases the abundance of 755 OR mRNAs and increases the abundance of 10 OR mRNAs. •, Significant decrease; +, no difference from littermate controls; ▴, increased abundance.

Fewer than 700 OR mRNAs meet our criteria for significant differences after the loss of either Lhx2 or Emx2 at the immature OSN stage. The loss of both homeodomain transcription factors at this stage reduces the abundance of 755 OR mRNAs and increases the abundance of 10 others (Fig. 6). This is not much more than an additive effect, arguing that Lhx2 and Emx2 lack epistasis and do not compensate for each other.

Among the Taar mRNAs, Taar2, Taar3, Taar4, Taar5, Taar6, Taar7a, 7b, 7d, 7e, 7f, and Taar9 are decreased. Only the mRNAs of the members of the Taar8 subfamily are not significantly affected.

**Figure 7.**
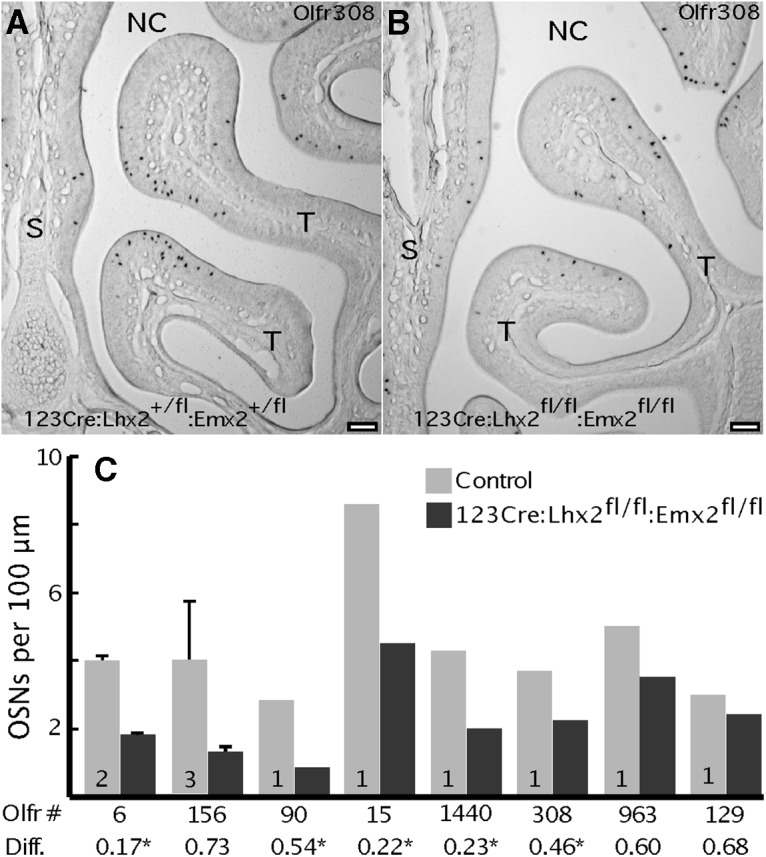
Conditional deletion of both *Lhx2* and *Emx2* in immature OSNs reduces OR expression frequencies. ***A***, *In situ* hybridization shows that *Olfr308* is expressed in scattered OSNs in the ventral olfactory epithelium. ***B***, In 123Cre:Lhx2^fl/fl^:Emx2^fl/fl^ mice, the frequency of OSNs expressing *Olfr308* appears to be reduced. NC, Air space of the nasal cavity; S, nasal septum; T, turbinate. Scale bars, 100 µm. ***C***, Normalized cell counts from *in situ* hybridization images show that ORs are less frequently expressed in 123Cre:Lhx2^fl/fl^:Emx2^fl/fl^ mice compared with littermate controls. Diff., Normalized fold differences from the microarray experiment. *Significant difference (p 0.01; n 4; FDR 1%).

As in all other mutant strains we tested, changes in OR mRNA abundance in 123Cre:Lhx2^fl/fl^:Emx2^fl/fl^ mice are due to changes in the frequency of OR gene expression (Fig. 7). Even some OR genes whose mRNAs did not reach significance in the microarray experiment, such as *Olfr156*, tend to show reduced frequencies of expression (Fig. 7*C*). OR genes that increase in expression frequency in Emx2-null mice, such as *Olfr90* and *Olfr15* (McIntyre et al., 2008), show reduced expression frequency when both Lhx2 and Emx2 are absent. These findings reinforce the conclusion that OR gene expression depends more directly and strongly on Lhx2 than Emx2.

### Zonal expansion suggests different roles for Lhx2 and Emx2

**Figure 8.**
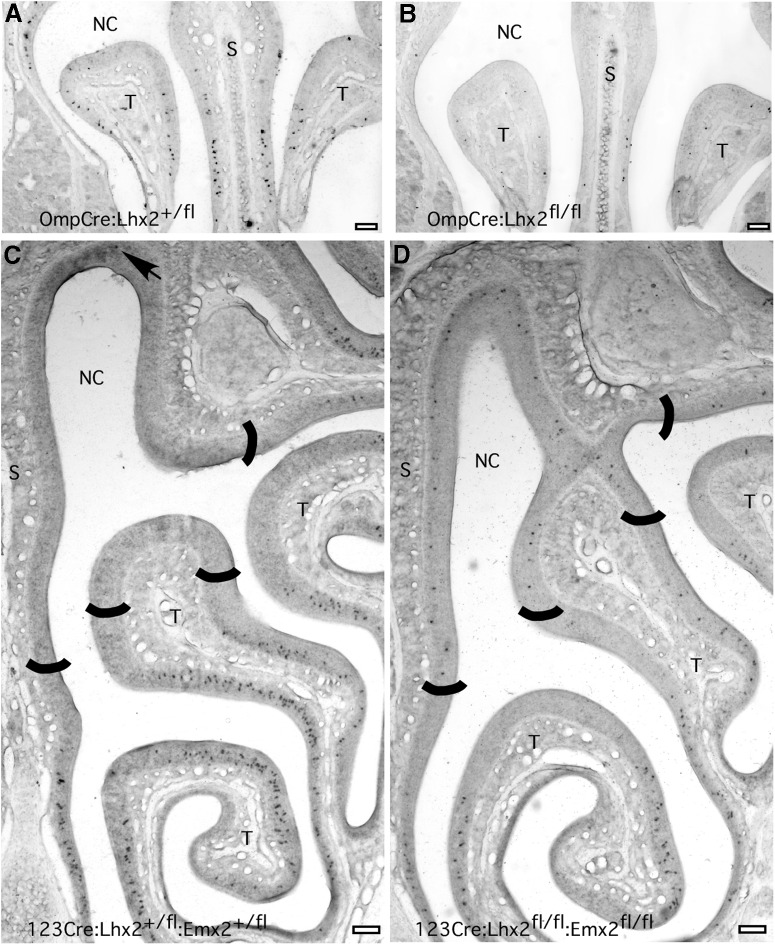
The absence of Lhx2 and the absence of Emx2 have distinctly different effects on *Olfr15* expression frequency. ***A***, *In situ* hybridization image showing normal *Olfr15* expression in the ventral region of the olfactory epithelium in a littermate control. ***B***, When *Lhx2* is deleted in OSNs, *Olfr15* expression frequency decreases. ***C***, An image of the left half of a more caudal section from a littermate control shows the normal expression of *Olfr15* in the ventral olfactory epithelium. This image shows one of the rare instances of the misexpression of *Olfr15* in the dorsal olfactory epithelium in control genotypes (arrow). ***D***, When both *Lhx2* and *Emx2* are deleted, *Olfr15* expression frequency decreases in the ventral region, where it is normally expressed, but misexpression increases in the dorsal region. The approximate boundaries between dorsal and ventral olfactory epithelium are marked in ***C*** and ***D***. NC, Air space of the nasal cavity; S, nasal septum; T, turbinate. Scale bars, 100 µm.

Conditional deletion of *Lhx2* or *Emx2* in OSNs almost never has effects on the zonal expression of ORs. *In situ* hybridization data for 11 OR mRNAs showed a shift in the zonal pattern in only one OR gene, *Olfr15*. We previously demonstrated that Olfr15 mRNA increases in embryos with germ-line deletions of *Emx2* (McIntyre et al., 2008). The increase is mostly due to increased expression frequency within the normal ventral expression zone of *Olfr15*, but the expansion of expression into the dorsal OR expression zone of the olfactory epithelium also contributes. We find this zonal expansion effect in all the conditional *Emx2* deletion strains tested. We note that *Olfr15* may be unusually susceptible to zonal misexpression. Across several dozen tissue sections from control littermates, we have observed three instances where a single dorsal zone OSN misexpresses *Olfr15*. The image shown in Figure 8*C* was chosen to illustrate one of these three instances.

In contrast to the increase in *Olfr15* expression frequency in both ventral and dorsal regions of the olfactory epithelium after the deletion of *Emx2*, when *Lhx2* is deleted in either immature or mature OSNs, *Olfr15* expression frequency is reduced (Fig. 8*A*,*B*). Olfr15 mRNA is also decreased when both *Lhx2* and *Emx2* are deleted, but *in situ* hybridization data reveal that this overall decrease obscures a zone-specific increase in *Olfr15* expression. In these double mutants, *Olfr15* expression is indeed decreased within its normal ventral expression zone, but the expansion of *Olfr15* expression into the dorsal zone that results from the loss of Emx2 is also apparent (Fig. 8*C*,*D*).

These findings suggest that Lhx2 and Emx2 play distinctly different roles in controlling OR expression, at least for some ORs. The absence of Lhx2 makes *Olfr15* less likely to be expressed, the same effect that the absence of Lhx2 has on other ORs. In contrast, the absence of Emx2 makes *Olfr15* more likely to be expressed, as if Emx2 helps to control the availability of *Olfr15* for expression.

### OSN turnover is unchanged in conditional *Lhx2* deletion mutants

**Figure 9.**
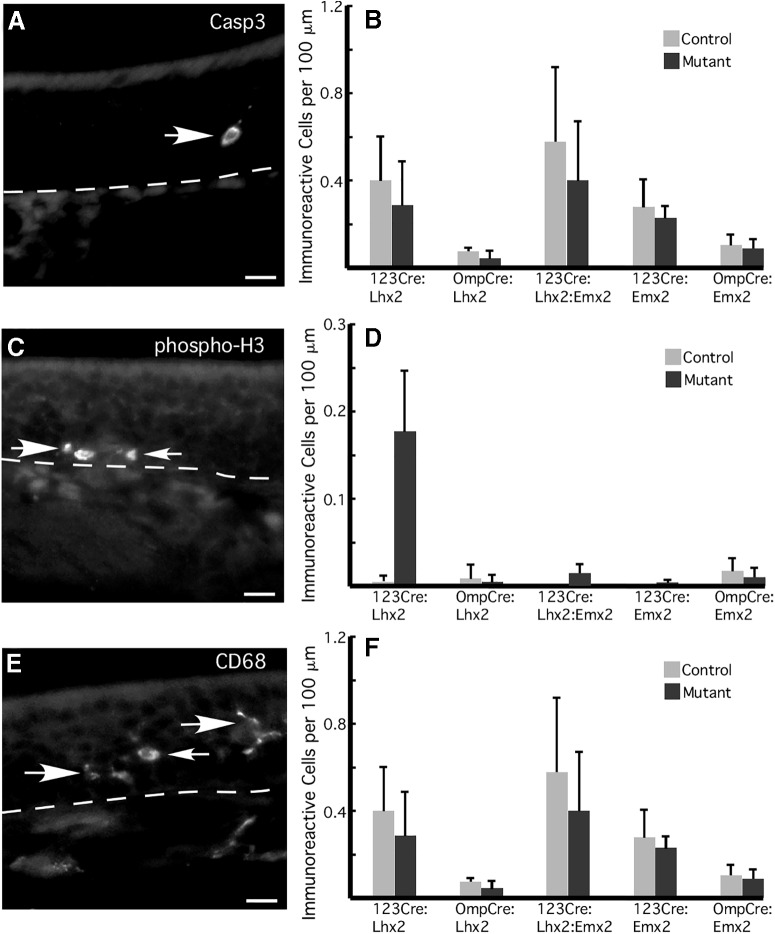
OSN turnover in Lhx2 and Emx2 mutant mice is indistinguishable from that of littermate controls. ***A***, Image of active caspase 3 immunoreactivity showing a cell undergoing apoptosis in the OSN layer of the olfactory epithelium (arrow). ***B***, Counts of the number of active caspase 3 immunoreactive OSNs reveal no significant differences between mutant mice and control littermates (*n* = 3 mice). ***C***, Phosphorylated histone 3 (phospho-H3) immunoreactivity in the basal cell layer identifies basal cells undergoing mitosis (arrows). ***D***, Quantification of phosphorylated histone 3 immunoreactive basal cells shows no significant differences between mutant mice and control littermates (*n* = 3 mice). ***E***, CD68 immunoreactivity identifies activated macrophages (arrows). ***F***, Quantification of CD68-immunoreactive macrophages in the olfactory epithelium reveals no significant differences between mutant mice and control littermates (*n* = 3 mice). Dashed lines, Location of the basal lamina. Scale bars, 50 µm.

Small reductions in mature OSN numbers accompany the reduced frequency of OR expression when *Lhx2* is deleted in OSNs, a finding that could indicate an alternative cause for altered OR expression. Increased turnover of OSNs could disrupt normal OSN development and lead to altered OR expression frequencies, similar to the effects seen at long survival times after unilateral naris occlusion or when olfactory neurogenesis is disrupted (Coppola and Waggener, 2012; Zhao et al., 2013). However, none of the strains show an increase in the frequency of cells immunoreactive for activated Casp3 (Fig. 9*A*,*B*). This evidence of normal OSN turnover is also supported by the lack of difference in the frequency of mitosis among basal cells (phosphorylated histone 3 immunoreactivity; Fig. 9*C*,*D*). Basal cell mitosis would increase if OSN turnover increased as tissue homeostasis mechanisms attempt to maintain normal numbers of OSNs. As is typical for juvenile mice reared in modern barrier housing facilities, most tissue sections show no cells immunoreactive for phosphorylated histone 3. Occasional sections show one or two dividing cells, and even more rare are sections with small clusters of dividing cells. The latter makes for substantial variation in cell counts. For example, the large difference in average counts between null mutants and littermate controls in 123Cre:Lhx2 mice (Fig. 9*D*) does not reach significance after correction for multiple testing (*q* = 0.3525; *n* = 3 mice).

Additional supporting evidence is the absence of an increase in CD68-immunoreactive macrophages in the olfactory epithelium (Fig. 9*E*,*F*). Macrophages respond to increased OSN turnover in order to help clear cell debris from the olfactory epithelium (Suzuki et al., 1995; Blomster et al., 2011; Heron et al., 2013).

In none of the five conditional deletion strains that we bred do we observe reduced numbers of immature OSNs. Instead, the absence of Lhx2 tends to cause small increases in immature OSNs in juvenile mice, contributing to a negative correlation between the numbers of mature and immature OSNs (*r* = 0.86). This is distinctly different from germ-line deletion of *Lhx2*, where both mature and immature OSNs are reduced (Kolterud et al., 2004). Overall, our data suggest that conditional deletion of *Lhx2* in OSNs leads to olfactory epithelia that have a slightly altered balance between numbers of immature and mature OSNs, but no detectable change in OSN turnover.

## Discussion

We assessed the effects of conditional deletion in OSNs of *Lhx2* and *Emx2* on OR expression. Unlike previous strategies that cause the loss of essentially all mature OSNs in *Lhx2* mutant mice (Hirota et al., 2007; Berghard et al., 2012), our strategy results in nearly normal numbers of OSNs, allowing us to reliably measure effects of the absence of Lhx2 on OR gene expression. We find that OR expression depends greatly upon Lhx2, and to a much lesser extent upon Emx2. Deleting *Lhx2* after OSN maturation is equivalent to the deletion of *Lhx2* that begins in immature OSNs, arguing that Lhx2 has the same role in regulating OR expression in both mature and immature OSNs. Emx2 may help to drive OR expression just like Lhx2, but it also has a distinctly different role that may involve controlling the availability of some OR genes for expression.

### Nearly all ORs and TAARs depend on Lhx2

All three conditional *Lhx2* deletion strains show the same effect, large decreases in the abundance of OR mRNAs far beyond what can be explained by small reductions in the number of mature OSNs. At the cellular level, this manifests as altered frequencies of OR expression.

Of the 1098 mRNAs encoding functional ORs that we measured, 965 are significantly decreased in at least one conditional *Lhx2* deletion strain. If we also include those OR mRNAs that displayed a tendency to differ (0.01 > *p* < 0.05), then only 44 OR mRNAs are consistently unaffected by the loss of Lhx2. Consistent with a critical role for Lhx2 in the expression of ORs, the ORs insensitive to the loss of Lhx2 tend to be expressed at low frequencies.

Loss of Lhx2 has similarly broad effects on TAAR gene expression. The mRNAs of all 13 functional TAAR genes expressed in OSNs are reduced in 123Cre:Lhx2^fl/fl^ mice, OmpCre:Lhx2^fl/fl^ mice, or both (but note that this is a tentative conclusion for Taar8a due to ambiguity in the probe set used to detect it). These data predict that TAAR genes have homeodomain sites in their enhancers, promoters, or both.

Importantly, the Lhx2 dependence of OR gene expression is almost certainly a direct effect. Lhx2 binds OR gene promoters and enhancers (Hoppe et al., 2003; Hirota and Mombaerts, 2004; Markenscoff-Papadimitriou et al., 2014). Our data provide no evidence for indirect effects, such as the reduced expression of other transcription factors, nor does the conditional deletion of *Lhx2* lead to a loss or turnover of OSNs that is sufficient to explain the strength and breadth of the effects observed. We conclude that Lhx2 is the transcription factor that acts at the homeodomain sites in the enhancers and proximal promoters of OR genes, and that nearly all OR genes require Lhx2 for expression.

Neither 123Cre nor OmpCre are able to recombine a Z/EG transgene or the native *Lhx2* locus at 100% efficiency, so OSNs expressing Lhx2-dependent OR genes in the conditional deletion Lhx2 mouse strains are mostly OSNs that continue to express Lhx2. We have not been able to directly identify OSNs that fail to express both Lhx2 and an OR or TAAR in these mutants, but because reductions in OR mRNA abundance far exceed the small reductions in mature OSN number, this must be the case. Given evidence that responding to odors and making the appropriate synaptic connections increases OSN lifespan (Schwob et al., 1992; Watt et al., 2004; Zhao et al., 2013), failure of OR expression should eventually reduce mature OSN numbers, consistent with our observation of fewer mature OSNs in *Lhx2* conditional deletion mice. The alternative explanation that haploinsufficiency of Lhx2 contributes to the effects we observe is not plausible because *Lhx2* heterozygotes expressing Cre do not show altered OR expression.

### Emx2 acts differently than Lhx2

In comparison with Lhx2, Emx2 is necessary for the normal expression of a much smaller fraction of OR genes, and *Emx2* deletion more often allows increased frequencies of OR expression; about 20–30% of the significantly affected OR mRNAs. In addition, the effects of deleting *Emx2* are greater in immature OSNs than in mature OSNs, which is not true of the Lhx2 mutant strains. When both *Lhx2* and *Emx2* are conditionally deleted in immature OSNs, no epistasis or compensatory effects are unmasked. These findings suggest that Lhx2 and Emx2 act mostly through independent mechanisms, an interpretation supported by the behavior of *Olfr15*. *Olfr15* expression frequency specifically decreases in the absence of Lhx2, while zonal misexpression of *Olfr15* is specific to the absence of Emx2. We cannot exclude the possibility that Emx2 acts like Lhx2 in driving the expression of some OR genes; but, if so, our data suggest that Emx2 also has a distinctly different role. We hypothesize that Emx2 helps to control the availability of OR genes for expression.

### OR expression in mature OSNs versus immature OSNs

By the time an OSN reaches maturity, it strongly expresses a single allele of one OR gene. Repressive chromatin modification might prevent the expression of any other OR allele, predicting that no OR genes should increase their expression in OmpCre:Lhx2^fl/fl^ mice. This seems inconsistent with our finding of 4 OR mRNAs that show significant increases and 56 additional OR mRNAs that show normalized fold differences of >1. At present, we are unable to rule out the possibility that mature OSNs can switch to a different OR allele (Shykind et al., 2004) if they lose the expression of their original OR allele. The small number and modest size of increases in OR expression could be explained by the rarity of opportunities for OR gene switching in OSNs lacking Lhx2, which are limited because nearly all OR genes depend on Lhx2 for expression. Another factor that could contribute to the limited opportunity for OR gene switching is the relative timing of OR gene choice and Cre recombination. The timing of OR gene choice is not yet firmly established, so it is possible that choice happens prior to the onset of Cre expression in immature OSNs in 123Cre:Lhx2^fl/fl^ mice. If true, this would explain the similar OR expression patterns between 123Cre:Lhx2^fl/fl^ mice and OmpCre:Lhx2^fl/fl^ mice.

Opportunities for differential survival of OSNs to cause increased frequency of expression of some ORs are limited because OSN turnover is not significantly elevated. However, we note that significant increases in the abundance of some Lhx2-independent OR mRNAs are detected at age P26 (OmpCre:Lhx2^fl/fl^ mice and 123Cre:Lhx2^fl/fl^:Emx2^fl/fl^ mice) but not at age P3 (123Cre:Lhx2^fl/fl^ mice), so it may be possible that better survival gradually leads to increased frequencies of OSNs that express Lhx2-independent ORs.

### Effects of altered OR expression on OSN maturation

Our data agree with previous evidence that the absence of Lhx2 slows immature OSN differentiation and even prevents the transition to the mature OSN stage (Berghard et al., 2012). Given that OSN turnover is not detectably altered in our conditional *Lhx2* deletion strains, differences in OSN maturation may contribute to differences in the numbers of OSNs present. In neonatal 123Cre:Lhx2^fl/fl^ mice, the numbers of mature OSNs are reduced slightly, while immature OSN numbers do not differ, which is consistent with the idea that immature OSNs are not producing mature OSNs as fast as their littermate controls. Because conditional deletion of *Emx2* does not affect OSN number, the slightly decreased numbers of mature OSNs and slightly increased numbers of immature OSNs in juvenile 123Cre:Lhx2^fl/fl^:Emx2^fl/fl^ mice must also be due to the absence of Lhx2. Immature OSN differentiation appears to be slowed in the absence of Lhx2, causing immature OSN numbers to build up postnatally. We conclude that the absence of Lhx2 halts OSN maturation by causing OR expression to fail, thereby preventing the feedback signaling that stabilizes the expression of a single OR allele and triggers the final maturation of the OSN (Dalton et al., 2013).

### The role of Lhx2 in OR gene choice

We find no evidence that Lhx2 is necessary for the zonal patterns of OR expression, nor for the singularity of OR expression per OSN. Instead, Lhx2 is necessary to drive OR expression. We envision two speculative models for Lhx2 action that are consistent with our data, one where Lhx2 has a direct role in OR gene choice, another where Lhx2 acts indirectly.

The ability of Lhx2 to bind OR enhancers, along with the evidence that OR enhancers form interchromosomal networks, raises the possibility that differing enhancer networks might determine which OR alleles become expressed (Markenscoff-Papadimitriou et al., 2014). Because Lhx2 binds these enhancers, Lhx2 is likely to help determine which enhancer networks form or which OR gene promoters interact with these networks, effects that could explain the Lhx2 dependence of OR gene expression frequencies. In the absence of Lhx2, the probability of the formation of each distinct enhancer network should decrease, probably to differing extents. This would lead directly to the differentially decreased expression of OR genes that we observe.

Alternatively, Lhx2 might indirectly contribute to OR gene choice. Epigenetic repression and derepression of OR alleles in immature OSNs might be balanced so as to randomly produce a fully derepressed allele at rare intervals. The role of Lhx2 in this model is to drive high levels of OR transcription when an OR allele becomes available, triggering the feedback that prevents the expression of any other OR allele. If OR genes differ in the degree to which their transcription depends on Lhx2 relative to other transcriptional regulators, then those OR genes that are most dependent on Lhx2 would have the lowest probability of becoming stably expressed and the most difficulty maintaining feedback repression in mature OSNs. This model would also result in the kinds of differences in OR expression frequency that we observe.

These two general models are not mutually exclusive and could both be active. They both explain how OR expression frequencies could have a fundamental randomness yet depend so strongly on Lhx2, even after OSNs have completed differentiation.

## Synthesis

The decision was a result of the Reviewing Editor Anna Menini and the peer reviewers coming together and discussing their recommendations until a consensus was reached. A fact-based synthesis statement explaining their decision and outlining what is needed to prepare a revision is listed below. The following reviewer(s) agreed to reveal their identity: Anna Berghard

The experimental design utilizes several powerful tools with the potential to address if Lhx2 and Emx2 transcription factors are important for expression of odorant receptor genes in olfactory sensory neurons (OSNs), starting from two different stages during the neuronal differentiation process.

However, several issues still remain, as detailed below.

1) The authors have to show that there indeed are OSNs without Lhx2 in the epithelium of the mouse lines where the gene should be deleted. There is no such data in the manuscript.

2) The difference in number of mature neurons between control and 123Cre:Lhx2fl/fl in Fig. 2 F - I does not agree with the difference in level of "mature OSN mRNAs" data mentioned in the text. Moreover, the thickness of the mature OSN layer is not a precise enough measurement and particularly as the cellularity in the epithelium obviously is different in the representative photos shown. As conventional Lhx2 and Msx2 knockouts has fewer differentiated OSNs the quantitative assessment of differentiated OSNs is a key point for the study. Given the inefficiency of the recombination by two different Cre-drivers all ISH analyses have to be corrected against counts of how many mature neurons are present e.g. per 100 micrometer unit in controls versus in conditional mutants.

3) It is known in the field that there is considerable cell death due to competition between OSNs during the first weeks of life. This mechanism thus is in operation also in the mutant epithelium that presumably is mosaic with regard to presence of Lhx2. This needs to be taken into consideration when interpretating the data.

4) The reader is left wondering where to find information which genes were in the array experiments that assessed expression of mature and immature OSN genes. And how was the data handled?

5) The experimental design never can show that e.g. Lhx2 directly regulates odorant receptor expression. Even if the Cre-driver line was not only conditional but also inducible, effects on expression of a given gene could still be indirect e.g. via "regulation of the regulator" (i.e. Lhx2 regulating expression of other genes controlling odorant receptor gene expression). The text misleads a reader that is not well read on gene regulation in an unacceptable way. Please modify the text.

6) The authors should be more conservative in their interpretation of the Emx2 conditional deletions. I have no issue with the partial deletion of Lhx2 because the phenotype is robust even under this partial deletion, but it is possible the Emx2 plays a more critical role in OR gene regulation that is masked by the incomplete nature of the conditional deletion.

7) Although e.g. Excel will produce standard deviations with only two input values this is wrong statistically. n needs to be at least 3 (really 4). Take away SD where number of animals was 2 (several bar graphs). Alternatively, in order to show assay variation, each 100 micrometer value could be regarded as one measurement point and SD shown for that. The number of animals, i.e. biological replicates, should still be as clear as it is now in the graphs.

8) The text that is underlined needs to be proof-read for language and terminology as it is non-interpretable in some instances.
